# Discovery of a Mammalian Splice Variant of Myostatin That Stimulates Myogenesis

**DOI:** 10.1371/journal.pone.0081713

**Published:** 2013-12-02

**Authors:** Ferenc Jeanplong, Shelley J. Falconer, Jenny M. Oldham, Mark Thomas, Tarra S. Gray, Alex Hennebry, Kenneth G. Matthews, Frederick C. Kemp, Ketan Patel, Carole Berry, Gina Nicholas, Christopher D. McMahon

**Affiliations:** 1 AgResearch Ltd, Hamilton, New Zealand; 2 School of Biological Sciences and Institute for Cardiovascular and Metabolic Research, University of Reading, Reading, United Kingdom; Charité Universit?tsmedizin Berlin, NeuroCure Clinical Research Center, Germany

## Abstract

Myostatin plays a fundamental role in regulating the size of skeletal muscles. To date, only a single myostatin gene and no splice variants have been identified in mammals. Here we describe the splicing of a cryptic intron that removes the coding sequence for the receptor binding moiety of sheep myostatin. The deduced polypeptide sequence of the myostatin splice variant (MSV) contains a 256 amino acid N-terminal domain, which is common to myostatin, and a unique C-terminus of 65 amino acids. Western immunoblotting demonstrated that MSV mRNA is translated into protein, which is present in skeletal muscles. To determine the biological role of MSV, we developed an MSV over-expressing C_2_C_12_ myoblast line and showed that it proliferated faster than that of the control line in association with an increased abundance of the CDK2/Cyclin E complex in the nucleus. Recombinant protein made for the novel C-terminus of MSV also stimulated myoblast proliferation and bound to myostatin with high affinity as determined by surface plasmon resonance assay. Therefore, we postulated that MSV functions as a binding protein and antagonist of myostatin. Consistent with our postulate, myostatin protein was co-immunoprecipitated from skeletal muscle extracts with an MSV-specific antibody. MSV over-expression in C_2_C_12_ myoblasts blocked myostatin-induced Smad2/3-dependent signaling, thereby confirming that MSV antagonizes the canonical myostatin pathway. Furthermore, MSV over-expression increased the abundance of MyoD, Myogenin and MRF4 proteins (P<0.05), which indicates that MSV stimulates myogenesis through the induction of myogenic regulatory factors. To help elucidate a possible role *in vivo*, we observed that MSV protein was more abundant during early post-natal muscle development, while myostatin remained unchanged, which suggests that MSV may promote the growth of skeletal muscles. We conclude that MSV represents a unique example of intra-genic regulation in which a splice variant directly antagonizes the biological activity of the canonical gene product.

## Introduction

Myostatin limits the size of skeletal muscles by inhibiting the proliferation and differentiation of muscle progenitors during development [1], [2]. The presence of myostatin in scallops, sea urchins and amphioxus indicates that it arose from a common ancestral gene about 900 million years ago [3]. While gene duplication events are thought to have given rise to multiple myostatin genes in bony fish, only one myostatin gene has been reported for mammals [4], [5]. Given the presence of myostatin before the emergence of chordates, it is interesting to note the lack of multiple myostatin genes in mammals, but this does not preclude the possibility that splice variants are present. Alternative splicing of pre-mRNA allows the generation of multiple, distinct mRNA transcripts and subsequently the production of structurally and functionally distinct proteins from a given gene, thus contributing to the high proteome diversity in mammals [6]. Splice variants of myostatin have been identified in crabs, fish, chickens and ducks, but have not been reported for mammals [7], [8], [9], [10]. Here we report the discovery of a novel myostatin splice variant (MSV) in sheep skeletal muscle that promotes myogenesis *in vitro*.

## Materials and Methods

### Animals

Cohorts of male New Zealand Romney sheep were weighed and then slaughtered at 1, 3, 6, 9, 12 and 18 months of age (n = 6 per age) after which the *semitendinosus* muscle was excised and weighed. A sample was collected from each muscle and snap frozen in liquid nitrogen and stored at −80°C for mRNA and protein analysis. *Semitendinosus* muscle from newborn Angus calfs and *gastrocnemius* muscle from mice (C57 strain) and rats (Sprague Dawley strain) were collected and snap frozen in liquid nitrogen, and then stored at −80°C for protein analysis. Primary myoblasts were isolated from fresh muscles collected from newborn lambs as described below. Polyclonal antibodies were raised in New Zealand White rabbits against the C-terminus of MSV. All animal experiments were carried out with the approval of the Ruakura Animal Ethics Committee, Hamilton, New Zealand.

### Isolation of Primary Sheep Myoblasts

Primary sheep myoblasts were isolated from hindlimb muscles of newborn lambs as we have described previously [11]. To verify the efficiency of myoblast isolation, we used immunocytochemistry to show that >90% of the myoblasts were positive for Pax7 (data not shown).

### Northern and Southern Blot Analysis

For Northern blot analysis, total RNA was isolated from sheep *semitendinosus* muscle with Trizol reagent (Invitrogen), and poly(A)^+^ RNA was purified with an mRNA purification kit (GE Healthcare) according to the manufacturer's instructions. Five micrograms of poly(A)^+^ RNA were separated on a 1.2% formaldehyde-agarose gel alongside with an RNA size ladder (Promega), and then transferred to an uncharged nylon membrane (GE Healthcare) by capillary action. A DNA probe was made using RT-PCR for exon 1 & 2 of sheep myostatin (GenBank accession number: AF019622, nt 1–621). Radioactive labeling of the probe and hybridization was carried out using conditions previously described [12]. In Southern blot analysis, fifteen micrograms of sheep genomic DNA were isolated as described elsewhere [13] and digested with restriction enzymes Bcl I, EcoR I or Hinc II (Invitrogen). The products were separated on a 1% agarose gel and then transferred to a positively charged nylon membrane (GE Healthcare). The membrane was hybridized with a radioactive probe as described above using conditions reported earlier [14]. The size and number of hybridization signals were compared with the expected size and number of restriction fragments of the sheep myostatin gene (GenBank accession number: DQ530260).

### Molecular Cloning of MSV

MSV cDNA was amplified by RT-PCR using flanking PCR primers around the ORF of myostatin cDNA. The forward primer (5′-TCAGACTGGGCAGGCATTAACG-3′, nt 3498–3519, GenBank accession number: DQ530260) was located in the 5′UTR and the reverse primer (5′-GCATATGGAGTTTTAAGACCA-3′, nt 9672–9692) in the 3′UTR. The PCR reaction was carried out with 2 µl of cDNA of sheep skeletal muscle as a template. Cycle conditions were 94°C for 2 min for pre-amplification denaturation, then 94°C for 30 sec, 55°C for 1 min, and 72°C for 2 min for 35 cycles. The PCR product was gel-purified and cloned into pGEM-T Easy *E. coli* plasmid vector according to the manufacturer's instructions (Promega). The insert of three clones was sequenced. A complete insert was assembled using VectorNTI software (Invitrogen) and aligned with sheep myostatin (GenBank accession numbers: AF019622 & DQ530260). Cattle MSV was similarly amplified by RT-PCR using ORF-specific primers for full length MSV, then cloned and sequenced.

### Production of MSV-specific Antibodies

Two polyclonal antibodies to MSV were produced. The first was to a synthetic oligopeptide (CYTPPYGQWIFHKERK aa 260-274 for MSVab) that was conjugated to keyhole limpet haemocyanin using the N-terminal cysteine by Auspep Pty Ltd (Australia). The second was to a recombinant protein for the C-terminal 65 amino acids of MSV (aa 257–321 for MSVab65), which was expressed and purified (see below). Two hundred micrograms of synthetic oligopeptide or recombinant protein for MSV were mixed with Freund's complete adjuvant and used to immunize rabbits, followed by two booster injections at four-week intervals with Freund's incomplete adjuvant. One week after the last boost, rabbits were terminally bled and serum was separated by centrifugation. Immunoglobulin was affinity purified from immune sera using Protein A agarose (Invitrogen).

### Recombinant Protein Expression in E. coli

The cDNA coding sequence was PCR amplified and cloned into the pET100/D-TOPO protein expression vector (Invitrogen) for the putative mature sheep MSV (rMSV, aa 275–321) and the entire novel C-terminus of sheep MSV (rMSV65, aa 257–321). Recombinant proteins were expressed in Origami B *E. coli* (Novagen), which promotes the formation of disulfide bonds, increased protein solubility and activity at room temperature [15]. Purification of recombinant proteins was carried out using the N-terminal His-tag following Invitrogen's native purification protocol and a Ni-NTA resin (Invitrogen). The purified recombinant protein was dialysed against two changes of dialysis buffer (20 mM TRIS-HCl pH 7.0, 150 mM NaCl) at 4°C overnight and any residual endotoxin was removed using a High-Capacity Endotoxin Removal Resin (Pierce/Thermo Scientific Inc.) following the manufacturer's protocol. The protein concentration was determined using the bicinchoninic acid protein assay (Sigma). The complete ORF of sheep MSV (aa 1-321) with a C-terminal V5-tag (rMSV-V5, pET101/TOPO vector, Invitrogen) was cloned into pcDNA3 plasmid and stably expressed in Chinese Hamster Ovary (CHO) cells. Serum-free (CD CHO AGT, Invitrogen) conditioned medium was harvested after seven days of incubation and purified on Ni-NTA resin under native conditions according to Invitrogen's purification protocol of recombinant proteins.

### Quantitative and Non-quantitative RT-PCR

Total RNA was extracted from cultured cells, skeletal muscles, heart, liver, brain, kidneys, testes, ovaries, gut, skin and aorta using Trizol reagent (Invitrogen) and 5 µg of total RNA was reverse transcribed into cDNA using the Superscript III Pre-Amplification kit (Invitrogen) according to the manufacturer's instructions. All PCR primers used in this study span across exon-exon boundaries to avoid the amplification of genomic templates (Table 1). PCR was carried out with 2.5 µl of a 1∶40 dilution of the reverse transcriptase reaction using the FastStart DNA Master plus SYBR Green I reagent on a LightCycler 2.0 PCR machine (Roche Diagnostics). A dilution series of pooled reverse transcriptase reactions was used to create a standard curve. The PCR products were separated in a 2.0% agarose gel and stained with SybrSafe (Invitrogen) to confirm their size. Representative PCR products were extracted from the gel and directly sequenced to confirm their identity. Data for each sample were normalized to the concentration of cDNA in each RT sample using Quant-it OliGreen ssDNA kit (Invitrogen) [16]. Tissue-specific expression of MSV and myostatin mRNA was determined in 20 µl PCR reaction mix using 1.0 µl RT reaction as a template, 400 nM of each primer, and 1 x PCR buffer (Roche Diagnostics), 0.2 mM of dNTPs (Invitrogen) and 1.0 U of FastStart Taq polymerase (Roche Diagnostics). The following cycling protocol was used: denaturation at 95°C for 2 min and amplification at 95°C for 15 sec, 55°C for 30 sec and 72°C for 1 min for 36 cycles. Representative PCR products were isolated from the gel, and their sequence identity was confirmed by direct sequencing.

**Table 1:** Oligonucleotide primers used in quantitative and non-quantitative PCR.

Gene name	Forward primer	Reverse primer	Accession number
Beta-actin	TCATCACCATCGGCAATGAG	TGTTGGCGTAGAGGTCTTTG	NM_001009784
GAPDH	TGCACCACCAACTGCTTAG	GATGCAGGGATGATGTTC	NM_008084
MSV-ORF	ATGCAAAAACTGCAAATCTTTG	TTATTTCATCCTAAAAGCTGCAGT	DL465814
MSV	GCTCAAACAACCTGAATCCAAC	CCATAGGGAGGAGTGTAAAAATG	DQ530260
Myostatin	GATCTTGCTGTAACCTTCCC	GTGGAGTGCTCATCACAATC	DQ530260

All oligonucleotide primer sequences are shown in 5′ to 3′ orientation.

### Development of an MSV Over-expressing Stable C_2_C_12_ Myoblast Line

The complete ORF of sheep MSV (aa 1–321) was cloned into the pcDNA3 mammalian expression vector which is driven by a constitutive cytomegalovirus promoter (Invitrogen). The plasmids either carrying the MSV coding sequence or the empty pcDNA3 vector were transfected into C_2_C_12_ myoblasts using the Lipofectamine 2000 reagent (Invitrogen) according to the manufacturer's protocol. A stable myoblast line constitutively over-expressing MSV (MSV-line) and an empty vector (Control-line) line were developed using 500 µg/ml of Geneticin (Invitrogen) selection. These stable myoblast lines did not undergo clonal selection to eliminate experimental artefacts stemming from unintended inactivation of genes in individual clonal lines. For each cell line, cells of the same passage number were used for all experiments, and maintained with continuous 500 µg/ml of Geneticin selection.

### Myoblast Proliferation Assays

Proliferation assays were performed on C_2_C_12_ mouse myoblasts and on primary myoblasts isolated from sheep including stable MSV over-expressing and control C_2_C_12_ lines. These cells were seeded onto uncoated 96-well tissue culture plates at a density of 1000 cells per well in DMEM medium with 10% fetal bovine serum (FBS) for C_2_C_12_ and 2000 cells per well for sheep myoblasts in DMEM medium with 20% FBS and 10% horse serum, and allowed to attach to the plate at 37°C in 5% CO_2_ overnight. C_2_C_12_ and sheep myoblasts were grown in DMEM medium with 5% FBS containing increasing concentrations of rMSV protein for 48 h (n = 8). The MSV over-expressing and control C_2_C_12_ lines were grown in DMEM medium with 5% FBS for 0, 24, 48 and 72 h (n = 8). Myoblast proliferation was determined using the WST-1 cell proliferation reagent (Roche Diagnostics) following the manufacturer's protocol. Each proliferation assay was run in the same plate and repeated two or three times.

### Smad2/3 Phosphorylation Assay

Myoblasts for the MSV- and Control-line were seeded onto 10 cm diameter tissue culture dishes at a density of 30,000 cells per cm^2^ in 10 ml of DMEM medium supplemented with 10% FBS and 500 µg/ml of Geneticin, and allowed to attach to the plate at 37°C in 5% CO_2_ overnight (n = 6 for each cell line). The following day, the plating medium was replaced with 5 ml of DMEM medium supplemented with 5% FBS and 500 µg/ml of Geneticin, and cells were allowed to adapt to the test medium for 6 h at 37°C in 5% CO_2_. The test medium was removed and complemented with recombinant myostatin protein (R&D Systems) at a concentration of 8 nM, and then the medium was quickly added back into the plates (n = 3 for each cell line). For the control plates (n = 3 for each cell line), the same volume of storage buffer without myostatin protein was added. Plates were incubated for 60 minutes at 37°C, 5% CO_2_, and then the test medium was removed and cells were harvested from the plate with 1.0 ml of ice cold lysis buffer (10 mM Hepes pH 7.9, 1.5 mM MgCl_2_, 10 mM KCl, 0.5% IGEPAL, 1 mM Na3VO4, 1 mM NaF and one Complete [Roche Diagnostics, Germany] protease inhibitor tablet per 50 mL buffer). Nuclear and cytoplasmic protein fractions were isolated from lysed cells following a protocol previously described [17]. The abundance of phosphorylated and total Smad2/3 proteins in the nuclear and cytoplasmic protein fractions were determined using Western immunoblotting.

### Western Immunoblotting

One hundred milligrams of *semitendinosus* muscle from sheep and from cattle, and a similarly sized sample of *gastrocnemius* muscle from mice and from rats were homogenized on ice in 1.0 mL of lysis buffer (see Smad2/3 phosphorylation assay). The homogenate was centrifuged at 10,000×*g* for 5 min at 4°C to remove tissue debris. The protein concentration was determined using the bicinchoninic acid (Sigma, MO) protein assay. Protein extracts were mixed in Laemmli sample buffer [18] and heated at 95°C for 5 min. Five (for nuclear protein fractions) or twenty (for cytoplasmic protein fractions or tissue protein extracts) micrograms of protein was separated on 10 or 15% SDS-PAGE gels, and then transferred to nitrocellulose membranes (BioTrace NT, PALL Corporation, FL) by electroblotting. All blots were stained with Ponceau S stain to confirm transfer and uniformity of loading. After washing in Tris buffered saline with 0.1% Tween 20 (TBST), the blots were blocked in blocking buffer (TBST buffer supplemented with 1% PVP-10, 1% PEG4000, 0.3% BSA fraction V, 0.01% Thimerasol) or with TBST buffer supplemented with 5% non-fat milk powder for one hour, and then incubated with different dilutions of primary antibodies: anti-MSVab, anti-myostatin [19], anti-CDK2 (sc-163, Santa Cruz Biotechnology), anti-Cyclin E (sc-481, Santa Cruz Biotechnology), anti-Myf5 (sc-302, Santa Cruz Biotechnology), anti-MyoD (sc-304, Santa Cruz Biotechnology), anti-MRF4 (sc-784, Santa Cruz Biotechnology), anti-MEF2 (sc-313, Santa Cruz Biotechnology), anti-Pax7 (sc-163, Santa Cruz Biotechnology), anti-Smad2/3 (sc-6032, Santa Cruz Biotechnology), anti-pSmad2/3 (Ser423/425, sc-11769R, Santa Cruz Biotechnology), anti-V5 (Invitrogen), anti-actin (A2066, Sigma), anti-SP-1 (sc-59, Santa Cruz Biotechnology) or anti-α-tubulin (T9026, Sigma) primary antibodies in the appropriate blocking buffer at 4°C overnight. After washes, membranes were incubated with the appropriate secondary antibody and developed with enhanced chemiluminescence.

### Surface Plasmon Resonance Analysis

These studies were performed using carboxymethylated dextran surfaces (CM5 chips) in a Biacore 3000 instrument (Biacore, Uppsala, Sweden). Approximately 2000 response units (RU) of recombinant myostatin (R&D Systems), activin receptor type IIB (ActRIIB, produced as a chimera of the extracellular domain of ActRIIB and Fc, R&D Systems) or rMSV was coupled to the surface of a flow cell using EDC/NHS (1-ethyl-3-{3-dimethylaminopropyl}-carbodiimide; N-hydroxy-succinimide) NH_2_ chemistry according to the manufacturer's standard protocols. Flow cell one served as an in-line reference to subtract bulk effects and any non-specific interactions. All samples were diluted in standard HBS-EP buffer (0.01 M HEPES, pH 7.4, 0.15 M NaCl, 3 mM EDTA, 0.005% Surfactant P20), which was also used as the running buffer. For kinetic analyses, the instrument was maintained at a constant temperature of 21°C and a flow rate of 30 µL/min throughout. A serial concentration series of rMSV, myostatin, ActRIIB and Fc (used as an internal negative control to subtract non-specific binding of ActRIIB-Fc chimera, R&D Systems) proteins was made, ranging from 6.25 nM to 100 nM, with the exception of the myostatin vs. myostatin analysis, where the concentration range was 62.5 nM to 1 µM (due to the significantly lower affinity for that interaction). For each concentration, association was measured over 180 s and dissociation was measured over a further 300 s. After each sample injection, protein samples were eluted and the surface regenerated with a 10 µL injection of 0.1 M NaOH.

### Myostatin Co-IP

Anti-MSV antibodies and normal rabbit IgG (DAKO), which served as a negative control, were conjugated to beads as previously described [20]. The anti-MSV antibodies were tested for their ability to immunoprecipitate rMSV-V5 in a pilot experiment. MSVab65 was selected because this antibody showed the highest efficiency in IP (data not shown). Total muscle protein was extracted from pooled *semitendinosus* muscles collected from three month old sheep (n = 3) using an extraction buffer (50 mM Tris-HCl pH 7.5, 100 mM NaCl, 0.1% [v/v] TritonX-100, Complete protease inhibitor [Roche Diagnostics]) on ice. Ten milliliters of total muscle protein extract was incubated with 100 µl of MSVab65 beads with and without rMSV-V5, or IgG beads on a rotating wheel at 4°C for 18 h. Beads were washed with ice cold extraction buffer four times including a last wash without TritonX-100. Immunoprecipitated proteins were eluted from beads with 200 µl of 0.1 M Na-citrate pH 2.5, neutralized with NaOH, mixed with 50 µl of 5 x Laemmli sample buffer and boiled for 5 min. Eluted protein samples were separated on a 15% SDS-PAGE gel, transferred to a nitrocellulose membrane and probed with an anti-myostatin antibody (sc-28910, Santa Cruz Biotechnology).

### CAGA-luciferase Reporter Assays

Myoblasts for the MSV- and Control-line were seeded onto uncoated 6-well tissue culture plates at a cell density of 10,000 cells/cm^2^. The following day myoblasts were transiently co-transfected with 3.0 µg of pGL3-(CAGA)_12_ firefly and 10 ng of pRL-TK renilla luciferase reporter plasmids [21] per well using Lipofectamine 2000 reagent (Invitrogen, CA) according to the manufacturer's protocol. Twenty-four hours following transfection, MSV- and Control-line myoblasts were treated either with 8 nM of recombinant myostatin (R&D Systems, n = 3 for each cell line) or with the storage buffer of myostatin without myostatin protein (n = 3 for each cell line) in DMEM medium with 5% FBS for 24 h. Myoblasts were harvested and luciferase activity was assayed with a Dual-Luciferase Reporter Assay System (Promega). Firefly luciferase luminescence values were normalized to renilla luciferase and expressed as fold induction to vehicle controls of the Control-line.

### Data Analysis

All data were analyzed by ANOVA using GenStat v13 software (VSN International Ltd). Post-hoc Student's t-tests were used to analyze data for the in vitro treatments. Data are presented as the mean ± standard error of the mean (S.E.M.).

## Results and Discussion

### Identification of MSV in Sheep Skeletal Muscle

**Figure 1:**
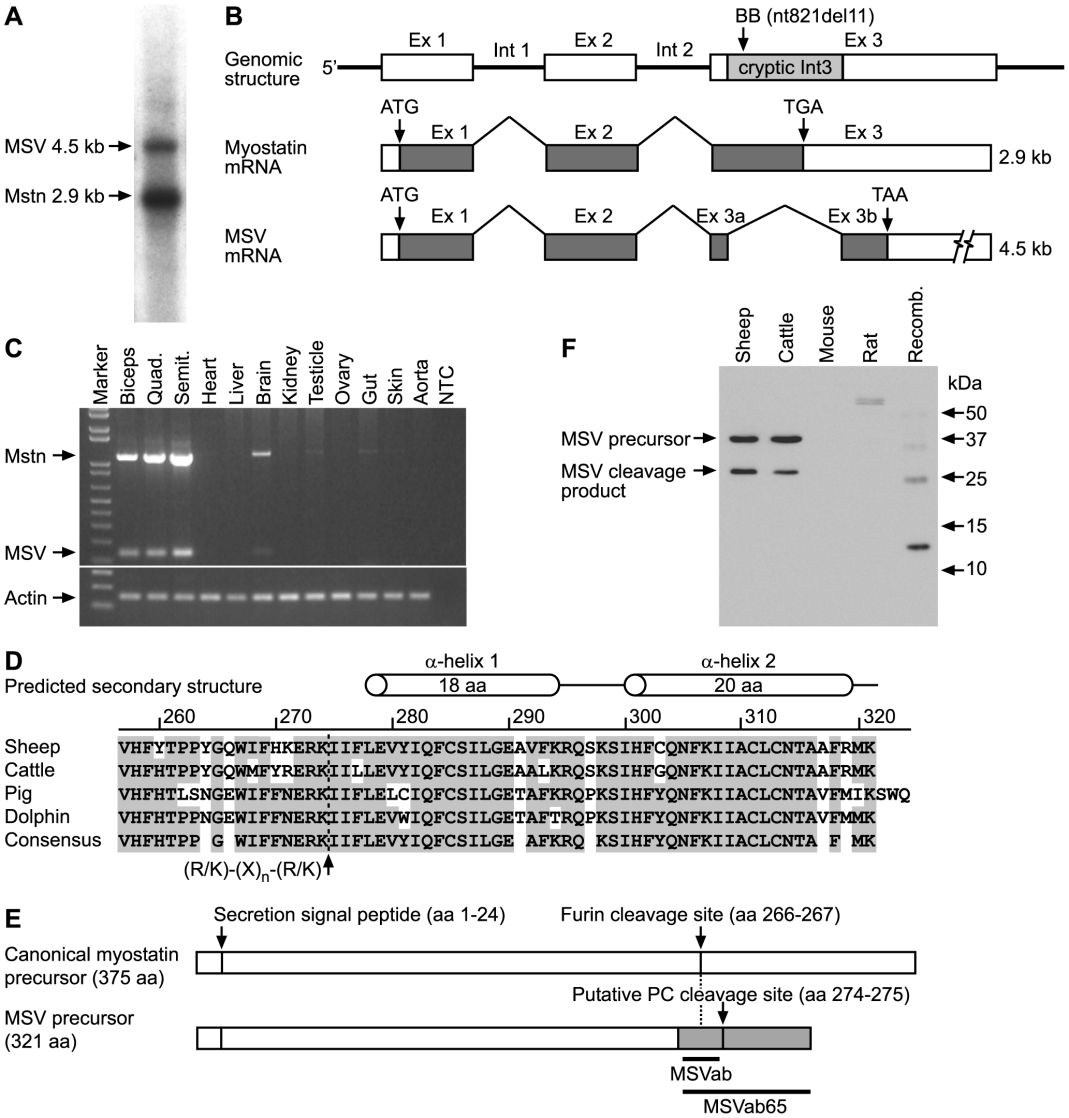
Alternative splicing of sheep myostatin pre-mRNA and translation of MSV mRNA into protein. (A) A representative Northern blot identified canonical myostatin (Mstn) and MSV mRNAs in poly(A)^+^ RNA isolated from sheep skeletal muscle using a radiolabeled probe complementary to exon 1 & 2 sequence of sheep myostatin (nt 1–621). (B) Schematic representation of alternative splicing of the myostatin gene. Genomic structure, splicing of canonical myostatin and MSV mRNAs are shown as determined by RT-PCR amplification and sequencing. The sheep myostatin gene has a cryptic third intron sequence (Int 3, 1011 bp) located 21 bp downstream of the intron 2/exon 3 boundary, thereby removing the coding sequence of the canonical mature myostatin protein. Alternate splicing creates a new ORF (966 bp) by appending a novel C-terminal coding sequence (exon 3b, 198 bp) to a truncated propeptide coding sequence of myostatin (exon 1 & 2 and 3a) in the MSV transcript. Open boxes show 5′ and 3′ untranslated regions, filled boxes represent translated sequences. Also shown are exons (Ex), introns (Int), translation start (ATG) and stop (TGA, TAA) sites, and the size of each transcript. Location of the 11 bp deletion in exon 3 identified in Belgian Blue cattle is also indicated. (C) Tissue-specific mRNA expression of MSV and myostatin was analyzed in *biceps femoris* (Biceps), *quadriceps* (Quad.) and *semitendinosus* (Semit.) muscles, and heart, liver, brain, kidney, testicle, ovary, gut, skin and aorta tissues of three months old sheep using RT-PCR. Actin was used as a positive control for each tissue sample. NTC is a no template PCR control. (D) Multiple polypeptide sequence alignment of the predicted C-terminus of MSV in sheep, cattle, pig and dolphin. A consensus proteolytic cleavage site [(K/R)-(X)_n_-(K/R)↓ where n = 0, 2, 4, 6 and X is any amino acid except cysteine at aa 271–274] has been identified for precursor convertases. A dotted line indicates the location of the putative cleavage site. The scale shows the positions of the amino acid residues in the MSV polypeptide sequence. The unshaded background highlights residues that are different from the consensus sequence. An *in-silico* predicted secondary structure of mature sheep MSV is also shown. (E) Schematic representation of the known and proposed proteolytic processing of canonical myostatin and MSV precursors, respectively. The location of the secretion signal peptide and the C-terminal cleavage sites are indicated. Grey filling shows the novel C-terminus of the MSV precursor. Black bars denote the location of polypeptide sequences used to raise MSV-specific polyclonal antibodies (MSVab and MSVab65). (F) Detection of MSV-immunoreactive proteins in *semitendinosus* muscles of sheep and cattle and its absence in *gastrocnemius* muscles of mouse and rat (20 µg of total protein per lane) using an anti-MSVab in Western immunoblotting. Recombinant peptide (Recomb.) corresponds to a polypeptide for the C-terminal 65 amino acids (11.9 kDa) of sheep MSV. Molecular weights of a protein marker are also indicated.

Northern blot analysis revealed two hybridization signals using a radio-labeled probe for exons 1 and 2 in sheep skeletal muscle (Figure 1A). The size of the lower band was consistent with canonical myostatin mRNA (2.9 kb) but the identity of the higher molecular weight band (4.5 kb) was unknown. We confirmed that this unknown mRNA species was transcribed from the myostatin locus and was not a pseudo-gene using Southern blot analysis (see Supporting Information Figure S1). Therefore, we hypothesized that the novel 4.5 kb mRNA was generated by alternative splicing of the myostatin pre-mRNA. To address this hypothesis, the cDNA sequence of the 4.5 kb product was determined by reverse transcription-polymerase chain reaction (RT-PCR), cloning and sequencing (GenBank accession number: DL465814). An alignment of the novel transcript with myostatin revealed a deletion of 1011 nucleotides within exon 3 of myostatin, due to the splicing of a cryptic third intron (Figure 1B). As a result, the coding sequence of the receptor binding moiety of myostatin (mature peptide), including the RSRR proteolytic cleavage site, was missing from the novel transcript. Instead, a 3′ terminal coding sequence was appended to a truncated 5′ propeptide coding sequence to create a novel ORF (Figure 1B). DNA sequence analysis identified consensus splicing donor and acceptor sites, a polypirimidine track and a branch point for the cryptic intron 3 sequence in sheep and other Cetartiodactyls (see Supporting Information Figure S2). We termed the new splice variant MSV. MSV mRNA was also identified in skeletal muscles of cattle using RT-PCR and sequencing (GenBank accession number: DL465818), but not in humans or rodents. To our knowledge, this is the first report of alternative splicing of myostatin in mammals. Thus, alternative splicing of myostatin pre-mRNA may be an uncommon event, but it has arisen independently in vertebrate and invertebrate species.

Alternative splicing can also generate unproductive mRNAs which are targeted for degradation through a mechanism called nonsense-mediated mRNA decay (NMD). NMD is activated when the ribosome encounters a premature termination (nonsense) codon >50 bases upstream of an exon/exon boundary [22]. It is unlikely that MSV mRNA is subjected to NMD because the cryptic intron is located in the last canonical exon of the myostatin gene and the splicing event does not result in a premature termination codon.

To determine the tissue-specific expression of MSV, we analyzed different tissues of sheep using RT-PCR. PCR primers flanking the cryptic intron 3 of MSV co-amplified MSV and myostatin cDNAs (Figure 1C). MSV was expressed predominantly in skeletal muscles (*biceps femoris, quadriceps and semitendinosus*) and was also present in brain at low abundance, but was undetectable in other tissues (Figure 1C).

### MSV is Translated into Protein and is Present in Skeletal Muscle

The deduced MSV polypeptide contains an N-terminal domain (aa 1–256) which is identical to the canonical myostatin propeptide, but has a novel C-terminal sequence of 65 amino acids (aa 257–321, Figure 1D & 1E, Supporting Information Figure S3). The presence of the signal peptide (aa 1–24) suggests that MSV is secreted in a similar manner to that of myostatin (Figure 1E) [2]. The N-terminal domain of MSV may function in a similar manner to the propeptide of myostatin by binding to the C-terminal, receptor binding moiety of myostatin to form an inactive latent complex [20]. Formation of this latent complex inhibited the interaction of the myostatin ligand with its cognate receptor, ActRIIB [23] and over-expression increased the muscle mass of transgenic mice [24], [25].

On close examination of the novel C-terminus of MSV, we identified an amino acid motif (KERK, aa 273–278), which contains a consensus site [(K/R)-(X)_n_-(K/R)↓ where n = 0, 2, 4, 6 and X is any amino acid except cysteine] for precursor convertases that may cleave and liberate the C-terminal peptide of 47 amino acids (aa 275–321) of MSV (Figure 1D & 1F) [26]. The RERK and NERK amino acid motifs of cattle, pigs and dolphins also fit with this consensus rule. Precursor convertases belong to an evolutionary conserved family of subtilisin-like, calcium-dependent serine proteinases that cleave pro-protein and pro-hormone precursors at paired basic amino acids to generate biologically active peptides [26]. To better understand the secondary structure of the C-terminus of sheep MSV, we subjected the polypeptide sequence to an *in silico* analysis (SSpro, Institute for Genomics and Bioinformatics, University of California, Irvine, Figure 1D). The analysis revealed two putative alpha helices in sheep and this was confirmed for cattle, dolphins and pigs. In support, phylogenetic analysis of the third exon of myostatin indicates that MSV may have only emerged in the Cetartiodactyla clade during evolution and may not be present in other mammals (see Supporting Information Figure S4) [27].

An MSV-specific antibody (MSVab) recognised recombinant MSV (rMSV65), which verifies its specificity (Figure 1F). We also noticed that weaker immunoreactive bands were also detected at about 24 and 36 kDa which are in agreement with the predicted sizes of a homo-dimer and a homo-trimer of rMSV65. These homo-polymers of rMSV65 are likely to be produced by the oxidation of some of the four cysteine residues which can form inter-chain disulfide bonds. The predicted sizes of MSV precursor, propeptide and mature polypeptides are 37, 28.7 and 5.4 kDa, respectively. MSVab identified two immunoreactive bands in sheep *semitendinosus* muscle using Western blotting which accords with the expected sizes of MSV precursor and propeptide (28.7 and 37 kDa, Fig. 1F). As predicted, the size of immunoreactive bands in cattle was identical to that of sheep and no immunoreactive bands were detected in mouse and rat muscles in which MSV is not present (Fig. 1F). We acknowledge that further studies are required to confirm the proteolytic processing site of the MSV precursor and the identity of cleavage products.

### Over-expression of MSV Stimulates Myoblast Proliferation Associated with an Increased Nuclear Abundance of CDK2 and Cyclin E Proteins

**Figure 2:**
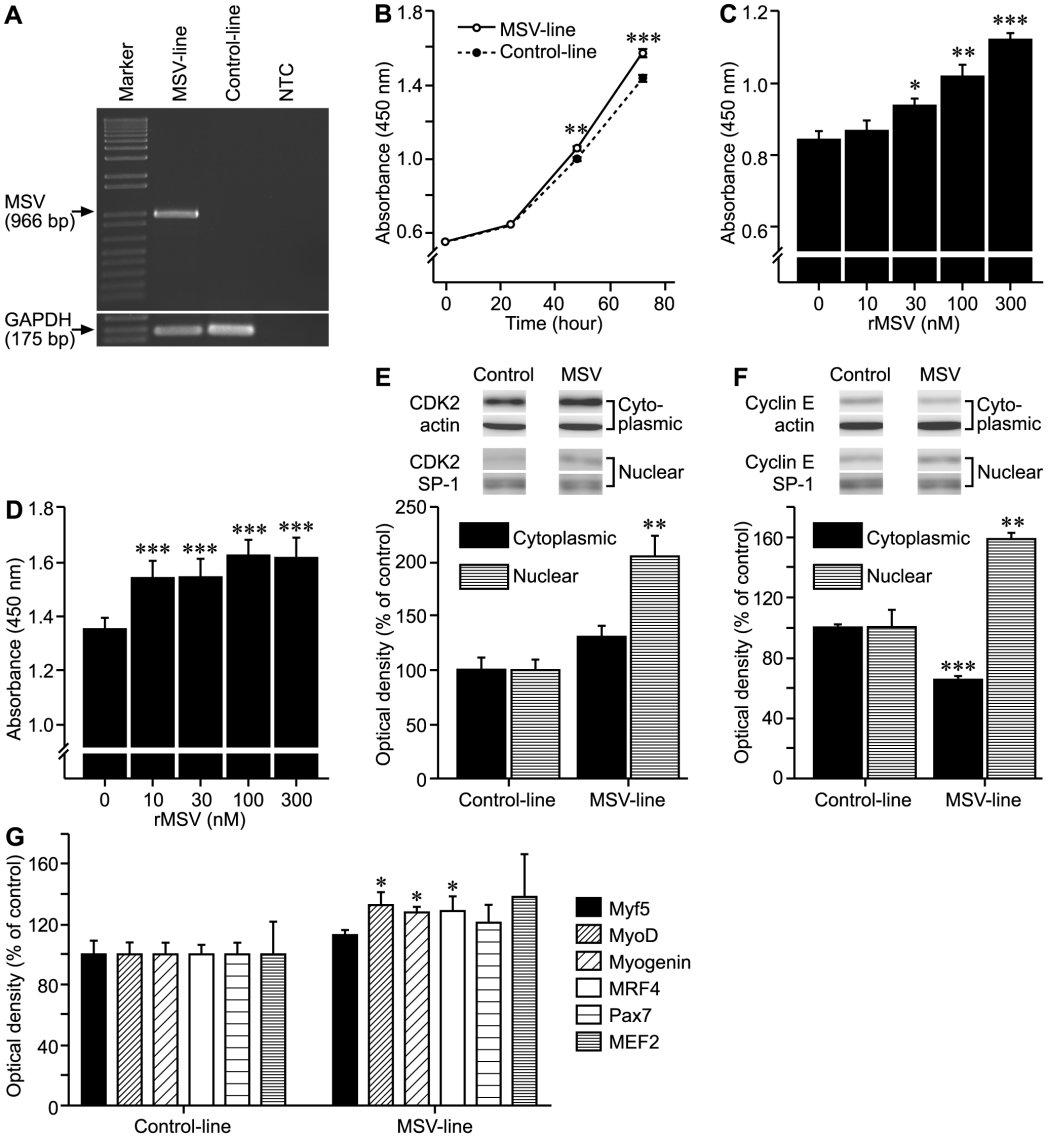
Functional analysis of MSV. (A) Detection of full length MSV mRNA in a stable MSV over-expressing (MSV-line) and an empty vector stably transfected C_2_C_12_ myoblast line (Control-line) using RT-PCR. GAPDH was used as a positive control for each sample. NTC is a no template PCR control. (B) Effect of endogenous over-expression of MSV on the proliferation of C_2_C_12_ myoblasts. Proliferation of the MSV- and Control-line was determined at 0, 24, 48 and 72 h using the WST-1 cell proliferation reagent (**P<0.01, ***P<0.001, n = 8). (C) Effect of rMSV on the proliferation of C_2_C_12_ myoblasts. C_2_C_12_ myoblasts were treated with increasing concentrations of rMSV for 48 h, and cell replication was determined using the WST-1 cell proliferation reagent (*P<0.05, **P<0.01, ***P<0.001, n = 8). (D) Effect of rMSV on the proliferation of sheep myoblasts. Sheep myoblasts were treated with increasing concentrations of rMSV for 48 h, and cell replication was determined using the WST-1 cell proliferation reagent (***P<0.001, n = 8). (E) The abundance of CDK2 protein in nuclear and cytoplasmic fractions of the MSV- and Control-line during proliferation (**P<0.01, n = 3). The abundance of actin and SP-1 proteins was used as cytoplasmic and nuclear loading controls, respectively. (F) The abundance of Cyclin E protein in nuclear and cytoplasmic fractions of the MSV- and Control-line during proliferation (**P<0.01, **P<0.001, n = 3). The abundance of actin and SP-1 proteins was used as cytoplasmic and nuclear loading controls, respectively. (G) The abundance of Myf5, MyoD, Myogenin, MRF4, Pax7 and MEF2 proteins was determined using Western immunoblotting in proliferating myoblasts of the MSV- and Control-line (*P<0.05, n = 3).

To determine the biological function of MSV, we developed a stable C_2_C_12_ myoblast line over-expressing full length sheep MSV with an empty vector transfected stable control line (Figure 2A). Proliferation of the MSV-line was greater than that of the Control-line (at least P<0.01, Figure 2B). While these results confirm that MSV stimulates myoblast proliferation, they could also be explained by the propeptide region, which is largely identical (explained above) to the myostatin propeptide, binding to and antagonizing the actions of myostatin [20]. To address whether or not the C-terminus of MSV could stimulate proliferation, recombinant protein was made for the C-terminal domain of MSV (rMSV). rMSV stimulated the proliferation of both murine C_2_C_12_ and sheep primary myoblasts in a dose-dependent manner (P<0.001, Figure 2C & 2D, respectively), which confirms that the C-terminal domain of MSV is bioactive. However, the relative contribution and roles of the N- and C-terminal domains to the biological function of MSV are unknown at present.

In support for a role of MSV in myoblast proliferation, the protein abundance of CDK2 and Cyclin E, key regulators of G1-S checkpoint of cell cycle, were increased in nuclear protein extracts from the MSV-line compared to that of the Control-line (P<0.05, Figure 2E & 2F). These observations are consistent with stimulated cell replication and raise the possibility that MSV may antagonize endogenous myostatin, which blocks the cell cycle at the G1-S checkpoint and down-regulates CDK2 [28], [29].

### Over-expression of MSV Stimulates the Expression of Myogenic Regulatory Factors

To better understand the effect of MSV on myogenesis, we measured the protein abundance of key muscle-specific transcription factors (Pax7, Myf5, MyoD, MRF4, Myogenin and MEF2) in the MSV- and Control-lines during proliferation. Endogenous over-expression of MSV increased the abundance of MyoD (P<0.05), Myogenin (P<0.05) and MRF4 (P = 0.058), but Pax7, Myf5 and MEF2 remained unchanged (Figure 2G). These data suggest that MSV functions, at least in part, via up-regulating MyoD, Myogenin and MRF4, which are critical transcription factors required for the execution of the myogenic program. The up-regulation of MyoD and myogenin by MSV is consistent with the blockade of endogenous myostatin, which inhibits myogenesis through the down-regulation of these transcription factors [28], [29], [30].

### MSV Binds to Myostatin

**Figure 3:**
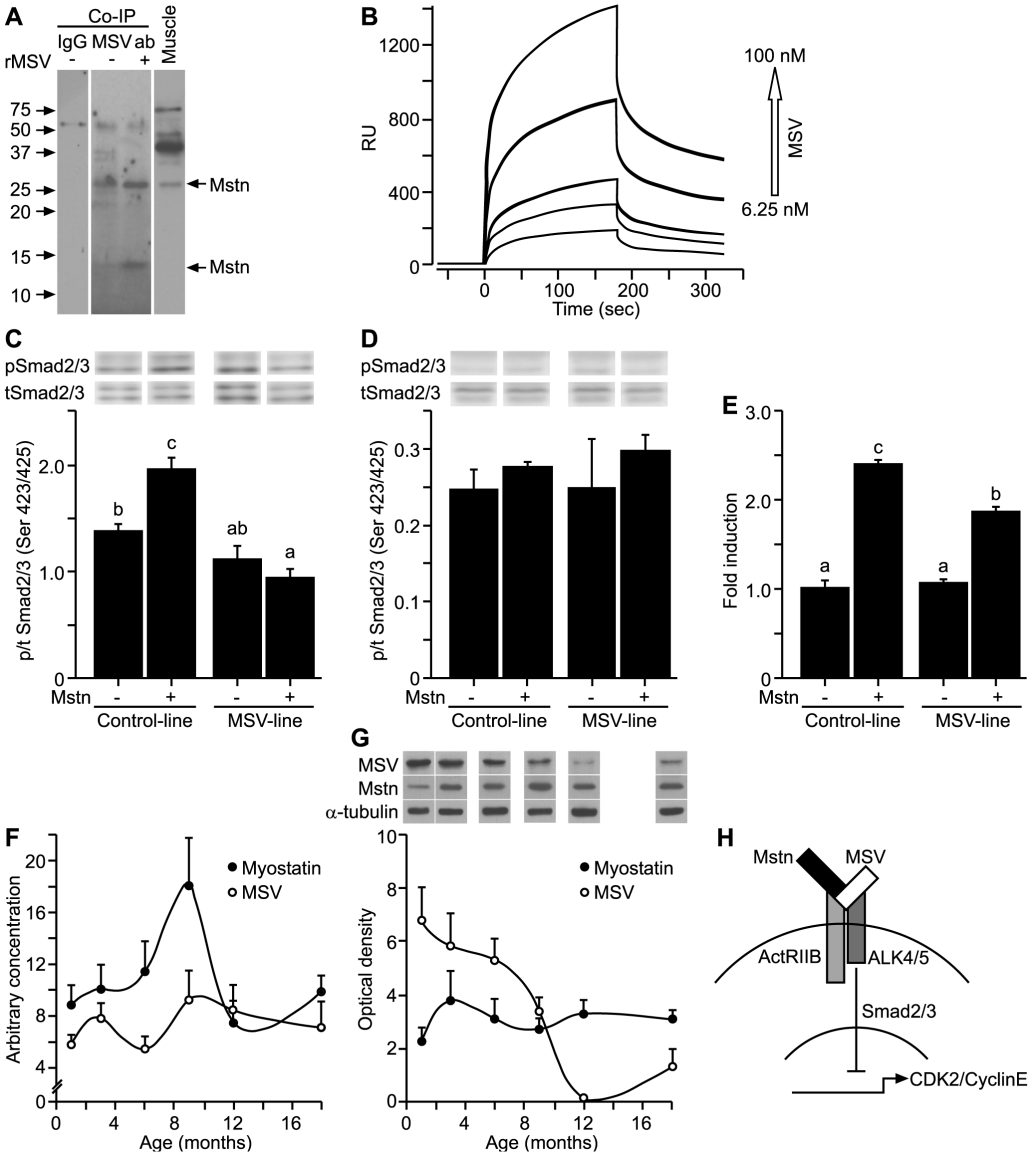
MSV binds to and antagonizes the canonical signaling of myostatin. (A) Co-immunoprecipitation (Co-IP) of myostatin protein was performed with an MSV-specific rabbit polyclonal antibody (MSVab) with normal rabbit IgG serving as a control (IgG) from a sheep muscle protein extract (Muscle). MSVab Co-IP was carried out with or without rMSV-V5 protein (rMSV). Protein samples were separated on a 15% SDS- PAGE, transferred to a nitrocellulose membrane and probed with an anti-myostatin antibody. Myostatin protein bands (Mstn) at 13 and 26 kDa are indicated with arrows. (B) Characterization of the interaction of mature MSV with mature myostatin using surface plasmon resonance assay. A representative sensorgram is shown for rMSV (6.25–100 nM) binding to and dissociating from myostatin immobilized on a sensorchip. For each concentration, association and dissociation were measured in duplicate and the response units (RU) are plotted against time. (C) Ratios of phosphorylated (Ser^423/425^) to total Smad2/3 protein abundance in nuclear protein fractions of proliferating myoblasts of the MSV-line and Control-line treated with or without myostatin (8 nM, n = 3). Unlike letters indicate significance (P<0.05). (D) Ratios of phosphorylated (Ser^423/425^) to total Smad2/3 protein abundance in cytoplasmic protein fractions of proliferating myoblasts of the MSV-line and Control-line treated with or without myostatin (8 nM, n = 3). (E) CAGA-luciferase reporter assay, wherein the MSV-line and Control-line were treated with or without myostatin (8 nM) for 24 hours (n = 3). Firefly luciferase luminescence values were normalized to renilla luciferase internal control and expressed as fold induction to vehicle controls of the Control-line. (F) Arbitrary concentrations (mean ± S.E.M.) of MSV and myostatin mRNAs in *semitendinosus* muscle of male sheep at 1, 3, 6, 9, 12 and 18 months of age (n = 6 per age) determined using qPCR. (G) The abundance (mean ± S.E.M.) of MSV (37 kDa) and myostatin (26 kDa) proteins during post-natal muscle growth in *semitendinosus* muscle of male sheep determined using Western immunoblotting at 1, 3, 6, 9, 12 and 18 months of age (n = 6 per age). α-tubulin (55 kDa) was used to assess the uniformity of loading. (H) A proposed model for the regulation of myoblast proliferation by MSV and myostatin (Mstn).

The observation that MSV stimulated the proliferation of myoblasts prompted us to investigate if MSV directly interacts with myostatin. We determined if an MSV antibody was able to co-immunoprecipitate (Co-IP) mature myostatin from protein extracts isolated from sheep skeletal muscle. A band corresponding to the 26 kDa mature myostatin dimer protein was detected using beads coated with MSV antibody, while no immunoreactive bands were evident when using control IgG beads (Figure 3A). The immunointensity of the 26 kDa mature myostatin band increased in Co-IP when the muscle protein extract was spiked with rMSV-V5 protein, which is consistent with MSV acting as a binding protein to pull down bound myostatin (Figure 3A). Furthermore, a faint 13 kDa band was also detected in Co-IP but not in skeletal muscle which may indicate the pull down of a low abundance monomeric form of mature myostatin protein, the expected size of which is 12.5 kDa (Figure 3A) [1]. These observations are in agreement with the high sequence identity between the N-terminal domain of MSV and the propeptide sequence of myostatin, which binds to myostatin [20], [23], [31]. However, the binding affinity of the novel C-terminal domain of MSV to myostatin remained unclear. To confirm a possible interaction, we employed a surface plasmon resonance assay. As a positive control, the binding of myostatin to ActRIIB was assessed. Myostatin bound to ActRIIB with high affinity as expected (Table 2). MSV peptide bound to myostatin protein with higher affinity than myostatin bound to itself (Table 2, Figure 3B). The calculated K_d_ for the myostatin/MSV interaction is comparable to the K_d_ of myostatin/propeptide, myostatin/follistatin or myostatin/FLRG interaction reported recently [32], [33]. This binding affinity suggests that the C-terminal domain of MSV directly interacts with myostatin, although as discussed earlier, the N-terminal domain of MSV is also likely to bind myostatin due to the similarity in sequence to the propeptide of myostatin. Therefore, MSV is a potential binding protein and antagonist of myostatin. If this is the case, MSV should block the molecular signalling of myostatin.

**Table 2:** Surface plasmon resonance kinetic analysis on the binding of MSV to myostatin, ActRIIB to myostatin and myostatin to itself.

Ligand$	Analyte	k_a_ (M^−1^s^−1^)	k_d_ (s^−1^)	K_D_ (M)
ActRIIB	Myostatin	4.01×e^4^	9.88×e^−4^	2.47×e^−8^
Myostatin	MSV	4.41×e^4^	4.32×e^−6^	9.79×e^−11^
Myostatin	Myostatin	5.58×e^3^	1.15×e^−3^	2.05×e^−7^

$ Immobilized onto the flow-cell. Rate constants of association and dissociation interactions (k_a_ and k_d_) and the equilibrium dissociation constants (K_D_) of Myostatin, ActRIIB and MSV interactions are shown.

### MSV Blocks Canonical Smad2/3-mediated Signalling of Myostatin

Endogenous over-expression of MSV inhibited the myostatin-induced increase of the ratio of phosphorylated to total Smad2/3^(S423/425)^ in nuclear protein extracts of C_2_C_12_ myoblasts, while myostatin treatment increased that ratio in the Control-line (Figure 3C). No change was detected for the ratio of phosphorylated to total Smad2/3^(S423/425)^ in the cytoplasmic protein extracts (Figure 3D). In support, myostatin-induced stimulation of a CAGA-luciferase reporter was antagonized in C_2_C_12_ myoblasts over-expressing MSV (MSV-line) to that of the Control-line suggesting that MSV can block the canonical signaling pathway of myostatin (Fig. 3E). We speculate that the partial blockade of the myostatin-induced stimulation of a CAGA-luciferase reporter is attributable to the expression level of MSV in the myoblast line. These results confirm that MSV protein functions as an antagonist of myostatin signalling.

### Expression of MSV Protein is Developmentally Regulated in Post-natal Muscles of Sheep

To further explore the role of MSV in post-natal growth of skeletal muscles, we measured the amount of MSV and myostatin mRNA and protein in *semitendinosus* muscles of male sheep from 1 to 18 months of age (Figure 3F & 3G). Concentrations of MSV mRNA did not show marked changes over time. In contrast, the concentration of myostatin transcripts increased in muscle from 1 to 9 months (P<0.05), then declined at 12 months of age (P<0.05), a point in development at which sheep reach adult size (Figure 3F and see Supporting Information Figs. S5A & S5B). The abundance of MSV precursor protein (37 kDa) was maximal at one month after birth and steadily declined to 12 months and slightly increased thereafter (P<0.001, Figure 3G). In contrast, the abundance of mature myostatin protein (26 kDa) did not alter markedly from 1 to 18 months of age (Figure 3G). We speculate that the greater abundance of MSV to that of mature myostatin protein may support the rapid growth of postnatal skeletal muscles by binding to and antagonizing the canonical signaling of myostatin thereby aiding hypertrophy of muscle fibres.

## Conclusions

In summary, we have identified a novel splice variant of myostatin in mammals. We propose a model wherein MSV protein binds to canonical myostatin which antagonizes Smad2/3-dependent myostatin signaling to increase the nuclear abundance of CDK2/Cyclin E complex (Figure 3H). An increase in the abundance of this complex promotes the G1-S phase transition of cells in the cell cycle resulting in enhanced myoblast proliferation. Our *in vitro* over-expression model indicated that beyond a positive effect of MSV on myoblast proliferation, MSV increased the protein abundance of key myogenic factors such as MyoD, Myogenin and MRF4 to promote myogenesis. Furthermore, the results of the Co-IP and surface plasmon resonance assay demonstrated that MSV binds myostatin and acts as a binding protein. Finally, MSV over-expression antagonized the canonical signalling of myostatin which suggests that MSV has the potential to regulate the bioactivity and/or bioavailability of myostatin to control muscle mass.

To the best of our knowledge, MSV represents a unique example of intra-genic regulation in biology where a splice variant is produced to directly control the bioactivity of the canonical gene product. This intriguing mechanism provides a direct way to influence the bioavailability of myostatin rather than relying on paracrine or endocrine control through the production of interacting proteins such as follistatin or FLRG [20], [24]. It would appear that there are numerous strategies to regulate myostatin and that some are species-specific. For example, male rodents have less mature myostatin than females and this was postulated to aid in development of sexual dimorphic growth of skeletal muscles [34]. However, we did not find a sexually dimorphic difference in the abundance of mature myostatin in skeletal muscles of sheep or in biopsy samples obtained from human subjects [35]. Therefore, we postulate that MSV has emerged through natural selection as a novel means to regulate the activity of myostatin in the Cetartiodactyla clade of mammals.

## Supporting Information

Figure S1 **Southern blot analysis of the sheep myostatin locus.** Fifteen micrograms of sheep genomic DNA was digested with restriction enzymes Bcl I, EcoR I or Hinc II, separated on a 1% agarose gel and transferred to a positively charged nylon membrane. The membrane was probed with α^32^P-dCTP labelled DNA probe homologous to exon 1 and 2 sequences of the sheep myostatin gene (nt 1-621, GenBank accession number: AF019622). Autoradiography shows positive hybridisation signals consistent with the predicted sizes of the DNA fragments (BclI 3004 and 3356 bp, EcoRI 990 and 5227 bp, HincII 5654 bp) determined by restriction site analysis of the sheep myostatin gene (GenBank accession number: DQ530260) using the Vector NTI software (Invitrogen). These results confirmed that there are no pseudo-genes for myostatin in the sheep genome.
(EPS)

Figure S2 **Alignment of the exon 3 DNA sequences of myostatin for cattle, dolphin, sheep and pigs.** Accession numbers are: AF320998 (GenBank) for cattle, ENSTTRG00000013877 (Ensembl) for dolphin, DQ530260 (GenBank) for sheep and AY208121 (GenBank) for pigs. Shown are the canonical splicing donor (GT) and acceptor (AG) sites, polypirimidine track (poly(Y)) and branch point (YNYTRAY, where Y is C or T; N is any; R is A or G nucleotides) of a cryptic intron 3 sequence. The unshaded background indicates nucleotides that are different from the consensus sequence. Numbering starts at the first nucleotide of exon 3 of canonical myostatin.
(EPS)

Figure S3 **Alignment of the polypeptide sequence of myostatin (Mstn) and MSV precursors in sheep.** The proteolytic processing sites for furin (RSRR) and the putative pre-protein convertases (KERK) are typed in bold. The divergence of MSV from myostatin and the N-terminal end of the putative mature MSV peptide are indicated with arrows.
(EPS)

Figure S4 **A phylogenetic topology plot showing the bootstrap consensus tree for a 1500 bp length of myostatin sequence from the start of exon 3 for all mammalian species for which there was complete sequence available in Ensembl (** http://www.ensembl.org **).** Numbers above each branch indicate the percent confidence for the division. Sequences were aligned using the ClustalW procedure and a phylogenetic tree constructed using the Maximum Composite Likelihood model in the Minimum Evolution method of MEGA version4 software (http://www.megasoftware.net). The branch point, donor and acceptor motifs are not present for splicing in marsupials and Xenarthrans, which suggests that MSV arose in placental mammals in the Boreoeutherian clade. In this group, two major clades are apparent based on the molecular classification system: Cetartiodactyla in which the splicing and translation of MSV have been confirmed, and in Primates where a putative single alpha helix of mature MSV is predicted by in silico analysis but no transcript has been confirmed. There is no discrepancy in the currently understood relationship among placental mammals using this region of myostatin to construct phylogeny (26). Incomplete splicing motifs (dogs, shrews, rat), or indels (horses, megabats, mice) either change the ORF (megabats), or introduce premature stop codons (horses, mice). This suggests that the splicing event became fixed in Cetartiodactyls (A), and may be present in Primates (B), but was lost in other branches. The gradient in the shading reflects this postulate. In support, fish, lizards, birds, frogs were also compared, but the splicing motifs were not present and, therefore, the data were excluded from this tree. The exception is alpacas where a single nucleotide deletion is present in the C-terminal coding sequence of MSV, which leads to a premature stop codon.
(EPS)

Figure S5 **Mean body mass ± S.E.M. (A) and** ***semitendinosus*** **muscle mass ± S.E.M. (B) at 1, 3, 6, 9, 12 and 18 months of age in male Romney sheep (n = 6).**
(EPS)

## References

[1] McPherron AC, Lawler AM, Lee SJ (1997) Regulation of skeletal muscle mass in mice by a new TGF-beta superfamily member. Nature 387: 83–90. 9139826 10.1038/387083a0

[2] Lee SJ (2004) Regulation of muscle mass by myostatin. Annu Rev Cell Dev Biol 20: 61–86. 15473835 10.1146/annurev.cellbio.20.012103.135836

[3] Xing F, Tan X, Zhang PJ, Ma J, Zhang Y, et al (2007) Characterization of amphioxus GDF8/11 gene, an archetype of vertebrate MSTN and GDF11. Dev Genes Evol 217: 549–554. 17551751 10.1007/s00427-007-0162-3

[4] Maccatrozzo L, Bargelloni L, Cardazzo B, Rizzo G, Patarnello T (2001) A novel second myostatin gene is present in teleost fish. FEBS Lett 509: 36–40. 11734202 10.1016/s0014-5793(01)03124-6

[5] McPherron AC, Lee SJ (1997) Double muscling in cattle due to mutations in the myostatin gene. Proc Natl Acad Sci U S A 94: 12457–12461. 9356471 10.1073/pnas.94.23.12457 PMC24998

[6] Black DL (2003) Mechanisms of alternative pre-messenger RNA splicing. Annu Rev Biochem 72: 291–336. 12626338 10.1146/annurev.biochem.72.121801.161720

[7] Covi JA, Kim HW, Mykles DL (2008) Expression of alternatively spliced transcripts for a myostatin-like protein in the blackback land crab, Gecarcinus lateralis. Comp Biochem Physiol A Mol Integr Physiol 150: 423–430. 18547854 10.1016/j.cbpa.2008.04.608

[8] Castelhano-Barbosa EC, Gabriel JE, Alvares LE, Monteiro-Vitorello CB, Coutinho LL (2005) Temporal and spatial expression of the myostatin gene during chicken embryo development. Growth Dev Aging 69: 3–12. 16180589

[9] Huang KL, Wang JW, Han CC, Liu HH, Li L, et al (2011) Developmental expression and alternative splicing of the duck myostatin gene. Comp Biochem Physiol Part D Genomics Proteomics 6: 238–243. 21592875 10.1016/j.cbd.2011.04.002

[10] Garikipati DK, Gahr SA, Roalson EH, Rodgers BD (2007) Characterization of rainbow trout myostatin-2 genes (rtMSTN-2a and -2b): genomic organization, differential expression, and pseudogenization. Endocrinology 148: 2106–2115. 17289851 10.1210/en.2006-1299

[11] McCroskery S, Thomas M, Maxwell L, Sharma M, Kambadur R (2003) Myostatin negatively regulates satellite cell activation and self-renewal. J Cell Biol 162: 1135–1147. 12963705 10.1083/jcb.200207056 PMC2172861

[12] Jeanplong F, Bass JJ, Smith HK, Kirk SP, Kambadur R, et al (2003) Prolonged underfeeding of sheep increases myostatin and myogenic regulatory factor Myf-5 in skeletal muscle while IGF-I and myogenin are repressed. J Endocrinol 176: 425–437. 12630927 10.1677/joe.0.1760425

[13] Montgomery GW, Crawford AM, Penty JM, Dodds KG, Ede AJ, et al (1993) The ovine Booroola fecundity gene (FecB) is linked to markers from a region of human chromosome 4q. Nat Genet 4: 410–414. 8401591 10.1038/ng0893-410

[14] Jeanplong F, Sharma M, Somers WG, Bass JJ, Kambadur R (2001) Genomic organization and neonatal expression of the bovine myostatin gene. Mol Cell Biochem 220: 31–37. 11451380 10.1023/a:1010801511963

[15] Lobel L, Pollak S, Lustbader B, Klein J, Lustbader JW (2002) Bacterial expression of a natively folded extracellular domain fusion protein of the hFSH receptor in the cytoplasm of Escherichia coli. Protein Expr Purif 25: 124–133. 12071707 10.1006/prep.2002.1618

[16] Lundby C, Nordsborg N, Kusuhara K, Kristensen KM, Neufer PD, et al (2005) Gene expression in human skeletal muscle: alternative normalization method and effect of repeated biopsies. Eur J Appl Physiol 95: 351–360. 16151837 10.1007/s00421-005-0022-7

[17] Marshall P, Chartrand N, Worton RG (2001) The mouse dystrophin enhancer is regulated by MyoD, E-box-binding factors, and by the serum response factor. J Biol Chem 276: 20719–20726. 11259421 10.1074/jbc.M102100200

[18] Laemmli UK (1970) Cleavage of structural proteins during the assembly of the head of bacteriophage T4. Nature 227: 680–685. 5432063 10.1038/227680a0

[19] Sharma M, Kambadur R, Matthews KG, Somers WG, Devlin GP, et al (1999) Myostatin, a transforming growth factor-beta superfamily member, is expressed in heart muscle and is upregulated in cardiomyocytes after infarct. J Cell Physiol 180: 1–9. 10362012 10.1002/(SICI)1097-4652(199907)180:1<1::AID-JCP1>3.0.CO;2-V

[20] Hill JJ, Davies MV, Pearson AA, Wang JH, Hewick RM, et al (2002) The myostatin propeptide and the follistatin-related gene are inhibitory binding proteins of myostatin in normal serum. J Biol Chem 277: 40735–40741. 12194980 10.1074/jbc.M206379200

[21] Dennler S, Itoh S, Vivien D, ten Dijke P, Huet S, et al (1998) Direct binding of Smad3 and Smad4 to critical TGF beta-inducible elements in the promoter of human plasminogen activator inhibitor-type 1 gene. EMBO J 17: 3091–3100. 9606191 10.1093/emboj/17.11.3091 PMC1170648

[22] Amrani N, Sachs MS, Jacobson A (2006) Early nonsense: mRNA decay solves a translational problem. Nat Rev Mol Cell Biol 7: 415–425. 16723977 10.1038/nrm1942

[23] Thies RS, Chen T, Davies MV, Tomkinson KN, Pearson AA, et al (2001) GDF-8 propeptide binds to GDF-8 and antagonizes biological activity by inhibiting GDF-8 receptor binding. Growth Factors 18: 251–259. 11519824 10.3109/08977190109029114

[24] Lee SJ, McPherron AC (2001) Regulation of myostatin activity and muscle growth. Proc Natl Acad Sci U S A 98: 9306–9311. 11459935 10.1073/pnas.151270098 PMC55416

[25] Yang J, Ratovitski T, Brady JP, Solomon MB, Wells KD, et al (2001) Expression of myostatin pro domain results in muscular transgenic mice. Mol Reprod Dev 60: 351–361. 11599046 10.1002/mrd.1097

[26] Seidah NG, Chretien M (1999) Proprotein and prohormone convertases: a family of subtilases generating diverse bioactive polypeptides. Brain Res 848: 45–62. 10701998 10.1016/s0006-8993(99)01909-5

[27] Springer MS, Murphy WJ, Eizirik E, O'Brien SJ (2003) Placental mammal diversification and the Cretaceous-Tertiary boundary. Proc Natl Acad Sci U S A 100: 1056–1061. 12552136 10.1073/pnas.0334222100 PMC298725

[28] Thomas M, Langley B, Berry C, Sharma M, Kirk S, et al (2000) Myostatin, a negative regulator of muscle growth, functions by inhibiting myoblast proliferation. J Biol Chem 275: 40235–40243. 10976104 10.1074/jbc.M004356200

[29] Joulia D, Bernardi H, Garandel V, Rabenoelina F, Vernus B, et al (2003) Mechanisms involved in the inhibition of myoblast proliferation and differentiation by myostatin. Exp Cell Res 286: 263–275. 12749855 10.1016/s0014-4827(03)00074-0

[30] Langley B, Thomas M, Bishop A, Sharma M, Gilmour S, et al (2002) Myostatin inhibits myoblast differentiation by down-regulating MyoD expression. J Biol Chem 277: 49831–49840. 12244043 10.1074/jbc.M204291200

[31] Wolfman NM, McPherron AC, Pappano WN, Davies MV, Song K, et al (2003) Activation of latent myostatin by the BMP-1/tolloid family of metalloproteinases. Proc Natl Acad Sci U S A 100: 15842–15846. 14671324 10.1073/pnas.2534946100 PMC307655

[32] Kondas K, Szlama G, Trexler M, Patthy L (2008) Both WFIKKN1 and WFIKKN2 have high affinity for growth and differentiation factors 8 and 11. J Biol Chem 283: 23677–23684. 18596030 10.1074/jbc.M803025200 PMC3259755

[33] Takehara-Kasamatsu Y, Tsuchida K, Nakatani M, Murakami T, Kurisaki A, et al (2007) Characterization of follistatin-related gene as a negative regulatory factor for activin family members during mouse heart development. J Med Invest 54: 276–288. 17878677 10.2152/jmi.54.276

[34] McMahon CD, Popovic L, Jeanplong F, Oldham JM, Kirk SP, et al (2003) Sexual dimorphism is associated with decreased expression of processed myostatin in males. Am J Physiol Endocrinol Metab 284: E377–381. 12388123 10.1152/ajpendo.00282.2002

[35] Oldham JM, Osepchook CC, Jeanplong F, Falconer SJ, Matthews KG, et al (2009) The decrease in mature myostatin protein in male skeletal muscle is developmentally regulated by growth hormone. J Physiol 587: 669–677. 19047209 10.1113/jphysiol.2008.161521 PMC2670088

